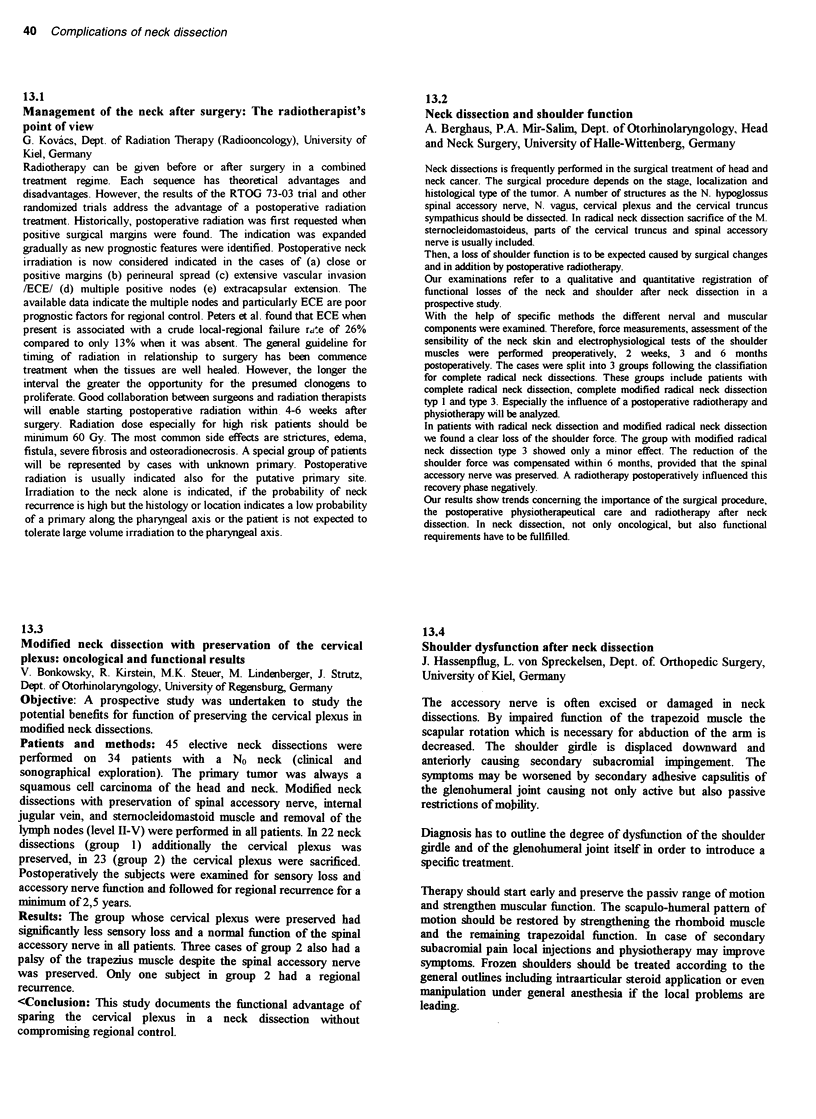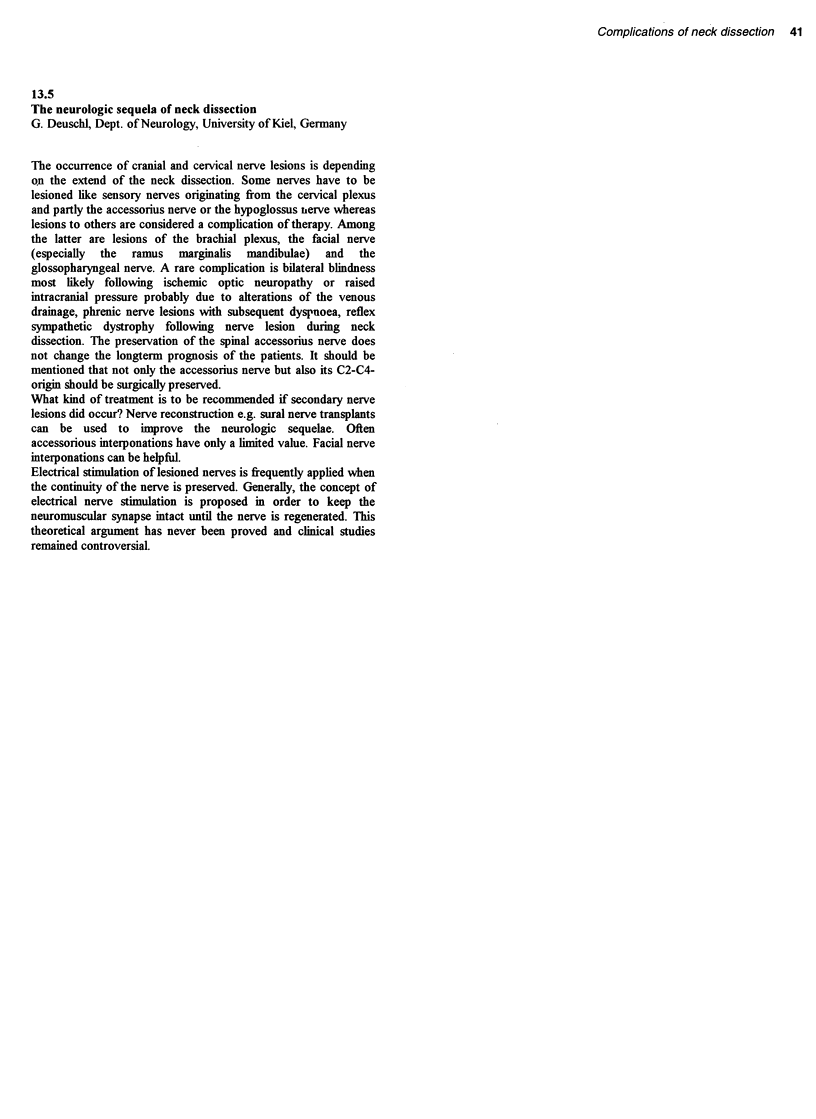# Complications of neck dissection

**Published:** 1998

**Authors:** 


### 13.1
### Management of the neck after surgery: The radiotherapist's point of view

G. Kovács, Dept. of Radiation Therapy (Radiooncology), University of Kiel, Germany

Radiotherapy can be given before or after surgery in a combined treatment regime. Each sequence has theoretical advantages and disadvantages. However, the results of the RTOG 73-03 trial and other randomized trials address the advantage of a postoperative radiation treatment. Historically, postoperative radiation was first requested when positive surgical margins were found. The indication was expanded gradually as new prognostic features were identified. Postoperative neck irradiation is now considered indicated in the cases of (a) close or positive margins (b) perineural spread (c) extensive vascular invasion /ECE/ (d) multiple positive nodes (e) extracapsular extension. The available data indicate the multiple nodes and particularly ECE are poor prognostic factors for regional control. Peters et al. found that ECE when present is associated with a crude local-regional failure rate of 26% compared to only 13% when it was absent. The general guideline for timing of radiation in relationship to surgery has been commence treatment when the tissues are well healed. However, the longer the interval the greater the opportunity for the presumed clonogens to proliferate. Good collaboration between surgeons and radiation therapists will enable starting postoperative radiation within 4-6 weeks after surgery. Radiation dose especially for high risk patients should be minimum 60 Gy. The most common side effects are strictures, edema, fistula, severe fibrosis and osteoradionecrosis. A special group of patients will be represented by cases with unknown primary. Postoperative radiation is usually indicated also for the putative primary site. Irradiation to the neck alone is indicated, if the probability of neck recurrence is high but the histology or location indicates a low probability of a primary along the pharyngeal axis or the patient is not expected to tolerate large volume irradiation to the pharyngeal axis.

### 13.2
### Neck dissection and shoulder function

A. Berghaus, P.A. Mir-Salim, Dept. of Otorhinolaryngology, Head and Neck Surgery, University of Halle-Wittenberg, Germany

Neck dissections is frequently performed in the surgical treatment of head and neck cancer. The surgical procedure depends on the stage, localization and histological type of the tumor. A number of structures as the N. hypoglossus spinal accessory nerve, N. vagus, cervical plexus and the cervical truncus sympathicus should be dissected. In radical neck dissection sacrifice of the M. sternocleidomastoideus, parts of the cervical truncus and spinal accessory nerve is usually included.
Then, a loss of shoulder function is to be expected caused by surgical changes and in addition by postoperative radiotherapy.
Our examinations refer to a qualitative and quantitative registration of functional losses of the neck and shoulder after neck dissection in a prospective study.
With the help of specific methods the different nerval and muscular components were examined. Therefore, force measurements, assessment of the sensibility of the neck skin and electrophysiological tests of the shoulder muscles were performed preoperatively, 2 weeks, 3 and 6 months postoperatively. The cases were split into 3 groups following the classifiation for complete radical neck dissections. These groups include patients with complete radical neck dissection, complete modified radical neck dissection typ 1 and type 3. Especially the influence of a postoperative radiotherapy and physiotherapy will be analyzed.
In patients with radical neck dissection and modified radical neck dissection we found a clear loss of the shoulder force. The group with modified radical neck dissection type 3 showed only a minor effect. The reduction of the shoulder force was compensated within 6 months, provided that the spinal accessory nerve was preserved. A radiotherapy postoperatively influenced this recovery phase negatively.
Our results show trends concerning the importance of the surgical procedure, the postoperative physiotherapeutical care and radiotherapy after neck dissection. In neck dissection, not only oncological, but also functional requirements have to be fullfilled.

### 13.3
### Modified neck dissection with preservation of the cervical plexus: oncological and functional results

V. Bonkowsky, R. Kirstein, M.K. Steuer, M. Lindenberger, J. Strutz, Dept. of Otorhinolaryngology, University of Regensburg, Germany

**Objective**: A prospective study was undertaken to study the potential benefits for function of preserving the cervical plexus in modified neck dissections.

**Patients and methods:** 45 elective neck dissections were performed on 34 patients with a $N_0$ neck (clinical and sonographical exploration). The primary tumor was always a squamous cell carcinoma of the head and neck. Modified neck dissections with preservation of spinal accessory nerve, internal jugular vein, and sternocleidomastoid muscle and removal of the lymph nodes (level II-V) were performed in all patients. In 22 neck dissections (group 1) additionally the cervical plexus was preserved, in 23 (group 2) the cervical plexus were sacrificed. Postoperatively the subjects were examined for sensory loss and accessory nerve function and followed for regional recurrence for a minimum of 2,5 years.

**Results:** The group whose cervical plexus were preserved had significantly less sensory loss and a normal function of the spinal accessory nerve in all patients. Three cases of group 2 also had a palsy of the trapezius muscle despite the spinal accessory nerve was preserved. Only one subject in group 2 had a regional recurrence.

<**Conclusion:** This study documents the functional advantage of sparing the cervical plexus in a neck dissection without compromising regional control.

### 13.4
### Shoulder dysfunction after neck dissection

J. Hassenpflug, L. von Spreckelsen, Dept. of Orthopedic Surgery, University of Kiel, Germany

The accessory nerve is often excised or damaged in neck dissections. By impaired function of the trapezoid muscle the scapular rotation which is necessary for abduction of the arm is decreased. The shoulder girdle is displaced downward and anteriorly causing secondary subacromial impingement. The symptoms may be worsened by secondary adhesive capsulitis of the glenohumeral joint causing not only active but also passive restrictions of mobility.

Diagnosis has to outline the degree of dysfunction of the shoulder girdle and of the glenohumeral joint itself in order to introduce a specific treatment.

Therapy should start early and preserve the passiv range of motion and strengthen muscular function. The scapulo-humeral pattern of motion should be restored by strengthening the rhomboid muscle and the remaining trapezoidal function. In case of secondary subacromial pain local injections and physiotherapy may improve symptoms. Frozen shoulders should be treated according to the general outlines including intraarticular steroid application or even manipulation under general anesthesia if the local problems are leading.

**13.5**
**The neurologic sequela of neck dissection**
G. Deuschl, Dept. of Neurology, University of Kiel, Germany

The occurrence of cranial and cervical nerve lesions is depending on the extend of the neck dissection. Some nerves have to be lesioned like sensory nerves originating from the cervical plexus and partly the accessorius nerve or the hypoglossus nerve whereas lesions to others are considered a complication of therapy. Among the latter are lesions of the brachial plexus, the facial nerve (especially the ramus marginalis mandibulae) and the glossopharyngeal nerve. A rare complication is bilateral blindness most likely following ischemic optic neuropathy or raised intracranial pressure probably due to alterations of the venous drainage, phrenic nerve lesions with subsequent dyspnoea, reflex sympathetic dystrophy following nerve lesion during neck dissection. The preservation of the spinal accessorius nerve does not change the longterm prognosis of the patients. It should be mentioned that not only the accessorius nerve but also its C2-C4-origin should be surgically preserved.
What kind of treatment is to be recommended if secondary nerve lesions did occur? Nerve reconstruction e.g. sural nerve transplants can be used to improve the neurologic sequelae. Often accessorious interponations have only a limited value. Facial nerve interponations can be helpful.
Electrical stimulation of lesioned nerves is frequently applied when the continuity of the nerve is preserved. Generally, the concept of electrical nerve stimulation is proposed in order to keep the neuromuscular synapse intact until the nerve is regenerated. This theoretical argument has never been proved and clinical studies remained controversial.